# Pinocembrin Reduces Keratinocyte Activation and Ameliorates Imiquimod-Induced Psoriasis-like Dermatitis in BALB/c Mice through the Heme Oxygenase-1/Signal Transducer and Activator of Transcription 3 Pathway

**DOI:** 10.1155/2022/7729836

**Published:** 2022-01-25

**Authors:** Kuo-Kuei Huang, Meng-Nan Lin, Hui-Chun Hsu, Yi-Ling Hsu, Ting-Ni Huang, I-Huang Lu, I-Hong Pan

**Affiliations:** Natural Medicine and Healthcare Technology Division, Biomedical Technology and Device Research Laboratories, Industrial Technology Research Institute, Hsinchu 30011, Taiwan

## Abstract

Psoriasis is an autoimmune disease characterized by chronic skin inflammation and excessive keratinocyte proliferation. The itchy, scaly, and erythematous lesions present on psoriatic skin negatively affect patients' quality of life. Pinocembrin is a flavonoid present in propolis, fruits, and vegetables. It exerts neuroprotective effects and was used for treating ischemic stroke in a human clinical trial. However, the effects of pinocembrin on psoriasis have never been examined. In this study, we evaluated the effects of pinocembrin on human HaCaT keratinocytes and BALB/c mice with imiquimod- (IMQ-) induced psoriatic dermatitis. In interferon-*γ*- (IFN-*γ*-) activated HaCaT cells, pinocembrin reduced the expression of inflammatory cytokines, namely, tumor necrosis factor-*α* (TNF-*α*), interleukin-6 (IL-6), and keratinocyte proliferation markers, namely, keratin (K)16, K17, and Ki-67. The mechanism underlying these inhibitory effects involved the regulation of the heme oxygenase- (HO-) 1/signal transducer and activator of transcription (STAT) 3 pathway. In the IMQ-induced psoriatic dermatitis mouse model, the topical application of pinocembrin significantly ameliorated the Skin Psoriasis Area and Severity Index score, epidermal thickness, inflammation, hyperplasia, hyperkeratosis, and cluster of differentiation (CD) 4^+^ T-cell infiltration. Expression of the inflammatory cytokines and keratinocyte proliferation markers in dorsal skin was significantly decreased in the pinocembrin-treated group. Meanwhile, in lesional skin, the expression of HO-1 was upregulated, but that of phospho-STAT3 (pSTAT3) was downregulated. Collectively, our results indicated the therapeutic potential of pinocembrin. Additional studies are warranted to evaluate its clinical benefits in patients with psoriasis.

## 1. Introduction

Psoriasis is a chronic autoimmune disease that causes skin lesions and systemic inflammation. The global prevalence of psoriasis is 2%–3% and it is equally distributed between men and women. Skin lesions are the predominant clinical manifestation of psoriasis. Inflamed, erythematous, and silvery scaly skin plaques are observed mainly on the elbows, knees, trunk, and scalp [1]. The relapsing inflammatory skin condition, comorbidities, and chronic medication can substantially affect patients' quality of life. A comparative study demonstrated that the quality of life of patients with psoriasis is as poor as that of patients with end-stage renal disease, visual disorders, and cancer [2]. Conventional drugs and treatment for moderate-to-severe psoriasis include cyclosporine, methotrexate, acitretin, and phototherapy. However, these treatments have limitations for long-term use because of their adverse effects and cumulative toxicity. Biologics demonstrating a higher efficacy and more favorable safety profile are a revolutionary treatment modality for psoriasis. However, concerns regarding the increased risks of malignancy and severe infection following the use of biologics such as tumor necrosis factor-*α* (TNF-*α*) inhibitors still exist [3]. Therefore, novel therapeutic interventions are required for patients with psoriasis.

Numerous studies have shown that the aberrant interaction between immune cells and epidermal keratinocytes is responsible for the initial inflammation and chronic lesions of the psoriatic skin [4]. In the context of immunity, although T-cells have an undisputed role in psoriasis, innate immune cells such as dendritic cells (DCs) and gamma delta T-cells also affect inflammatory cytokines expression and Th17 cells activation [5–7]. The aberrant proliferation and differentiation of keratinocytes are the hallmark features of psoriasis. Psoriatic keratinocytes can activate neutrophils, plasmacytoid dendritic cells, and T-cells [8–10]. Substantial proliferation and differentiation of keratinocytes are the consequences of responses to inflammatory cytokines including interferon-*γ* (IFN-*γ*), TNF-*α*, interleukin-22 (IL-22), and IL-17 [11]. During the uncontrolled differentiation of psoriatic keratinocytes, the fibrous cytoskeletal protein of keratin is changed from keratin (K)5 and K14 to K16 and K17, respectively. K17 is closely associated with Th1 response, and K17 is an antigen recognized by autoreactive T-cells [12, 13]. Under the inflammatory cytokine milieu, keratinocytes amplify the inflammation circuit by producing cytokines, chemokines, and proliferation-stimulating factors such as TNF-*α*, IL-17, IL-6, thymic stromal lymphopoietin, chemokine (C-X-C motif) ligand 8, and vascular endothelial growth factor [14, 15].

Heme oxygenase-1 (HO-1) is a stress-induced enzyme involved in the catabolism of heme in hemoglobin. Furthermore, oxidative metabolites, namely, biliverdin, carbon monoxide, and ferrous ion, contribute to protective effects such as anti-inflammatory and antioxidative effects [16]. Among several transcription factors involved in the molecular modulation of HO-1, nuclear factor erythroid 2-related factor 2 (Nrf2) is the critical one [17]. Accumulating evidence has indicated that HO-1 is involved in the inflammation of psoriatic skin, and HO-1 may be a therapeutic target in psoriasis treatment [18]. Under oxidative stress, keratinocytes are protected from damage through the induction of HO-1. Increased HO-1 expression was observed in psoriatic skin lesions. In addition, ultraviolet radiation treatment increased HO-1 expression in psoriatic skin. In animal studies, enhanced HO-1 expression in keratinocytes ameliorated cutaneous lesions in guinea pig and mouse models of psoriasis [18, 19]. Signal transducer and activator of transcription 3 (STAT3) is a transcription factor that conveys signals from cytokines or growth factors to the nucleus and activates downstream genes. Aberrant STAT3 activation was observed in main cell types involved in the pathogenesis of psoriasis including Th17 cells, *γδ* T-cells, and keratinocytes [20]. Furthermore, the abnormal proliferation and differentiation of keratinocytes stimulated with TNF-*α*, IL-6, and IL-22 were mediated by the STAT3 pathway [21].

Pinocembrin is a natural flavonoid isolated from various plants and propolis. Numerous in vitro and in vivo studies have demonstrated various pharmacological activities of pinocembrin including antibacterial, anti-inflammatory, and neuroprotective activities [22, 23]. The extract of the plant *Lippia graveolens* containing pinocembrin exhibited photochemopreventive activity under UVB radiation in mice with dermatitis [24]. Moreover, unlike other flavonoids, pinocembrin has been demonstrated to be safe for humans, and a phase II human trial of pinocembrin for ischemic stroke is ongoing in China [25, 26]. To date, no study has investigated the biological effects of pinocembrin on psoriasis. In this study, we evaluated the therapeutic potential of pinocembrin in psoriasis and explored the underlying mechanism of action. Our results demonstrated that pinocembrin inhibited IFN-*γ*-induced activation in human keratinocyte HaCaT cells. Furthermore, pinocembrin alleviated the severity of skin inflammation in imiquimod- (IMQ-) induced psoriatic dermatitis in mice, and the inhibitory effect may involve the HO-1/STAT3 pathway. Collectively, our data indicated the therapeutic potential of pinocembrin; thus, it can be further developed as an intervention for psoriasis treatment.

## 2. Materials and Methods

### 2.1. Chemicals and Reagents

Dulbecco's Modified Eagle Medium (DMEM), fetal bovine serum (FBS), antibiotics, trypsin-EDTA, sodium pyruvate, and Nonessential Amino Acids Solution were purchased from Gibco Life Technologies (Grand Island, NY, USA). Thiazolyl blue tetrazolium bromide (MTT), H_2_O_2_, sodium bicarbonate, 2-mercaptoethanol, and pinocembrin were purchased from Sigma (St. Louis, MO, USA). IFN-*γ*, IL-6, and prostaglandin E2 (PGE2) enzyme-linked immunosorbent assay (ELISA) kits were purchased from R&D systems (Minneapolis, MN, USA). Lipofectamine RNAiMAX reagent was purchased from Invitrogen (Carlsbad, CA, USA). Aldara cream (5% IMQ) was purchased from 3M Pharmaceuticals (Leicestershire, UK). Olivem 1000 was purchased from Essence Plus Co., Ltd. (Hsinchu, Taiwan). The RNeasy mini kit and QuantiTect reverse transcription kit were purchased from Qiagen (Hilden, Germany). The iQ SYBR Green Supermix and Alamar Blue were purchased from Bio-Rad (Hercules, CA, USA). A protease inhibitor was purchased from Abcam (Cambridge, MA, USA). The nuclear protein extraction kit used was purchased from Biotools Co., Ltd. (New Taipei City, Taiwan). The bicinchoninic acid (BCA) protein assay kit used was purchased from Thermo Scientific (Rockford, IL, USA). NuPAGE 4%–12% Bis-Tris gel was purchased from Invitrogen. Nitrocellulose membranes were purchased from Amersham Biosciences (Sunnyvale, CA, USA). Cell lysis buffer, phospho-STAT3 (pSTAT3) ((S727) #9134), STAT3 ((124H6) #9139), K17 ((D73C7) #4543), HO-1 ((P249) #5061), Nrf2 ((D1Z9C) #12721), Lamin B1 ((D4Q4Z) #12586), and *β*-actin ((8H10D10) #3700) primary antibodies and horseradish peroxidase-conjugated secondary antibodies (anti-rabbit IgG #7074 and anti-mouse IgG #7076) were purchased from Cell Signaling Technology (Beverly, MA, USA). Rabbit anti-mouse CD4 monoclonal (ab183685), rabbit anti-mouse CD8 monoclonal (ab237723), rabbit anti-mouse Ki-67 polyclonal (ab15580), and goat anti-rabbit IgG H&L (HRP) antibodies (ab6721) were purchased from Abcam (Cambridge, UK). The Immobilon Western chemiluminescent HRP substrate used was purchased from Millipore (Bedford, MA, USA).

### 2.2. Effects of Pinocembrin on HaCaT Cells

A human keratinocyte cell line, HaCaT, was purchased from the American Type Culture Collection and maintained in DMEM supplemented with 10% heat-inactivated FBS and antibiotics (100 U/mL penicillin G and 100 *μ*g/mL streptomycin) at 37°C in a humidified incubator containing 5% CO_2_ and 95% air. HaCaT cells were seeded in a 6-well plate at a density of 2 × 10^5^ cells/well overnight, treated with various concentrations of pinocembrin for 6 h, and then stimulated with IFN-*γ* for 18 h (for the analysis of inflammatory cytokines) or 48 h (for the analysis of proliferation markers). Secreted IL-6 and PGE2 were detected using commercial ELISA kits, and cell viability was determined using the MTT method. Total RNA was isolated using the RNeasy mini kit according to the manufacturer's instructions.

### 2.3. siRNA Transfection

HaCaT cells were seeded in a 6-well plate at a density of 2 × 10^5^ cells/well overnight and transiently transfected with HO-1 siRNA for 6 h by using the Lipofectamine RNAiMAX reagent according to the manufacturer's protocol. The cells were then incubated in fresh media with 10% FBS for further manipulation.

### 2.4. Western Blot Analysis

HaCaT cell lysates were prepared using cell lysis buffer containing protease inhibitor. Nuclear protein extracts were prepared using the nuclear protein extraction kit. Mouse skin tissues were homogenized in lysis buffer by using a hand-held homogenizer. Total proteins from extracts were quantified using the BCA protein assay kit. Equal amounts of proteins were subjected to electrophoresis in the NuPAGE 4%–12% Bis-Tris gel, transferred onto nitrocellulose membranes, blocked, and incubated with primary antibodies against pSTAT3, K17, HO-1, Nrf2, STAT3, Lamin B1, and *β*-actin at 1 : 1000 diluted in TBST with 5% skimmed milk. Subsequently, the membranes were incubated with horseradish peroxidase-conjugated secondary antibodies at 1 : 5000 diluted in TBST with 5% skimmed milk, and the signal intensities of target proteins were detected using the Immobilon Western chemiluminescent HRP substrate. The intensities of target proteins were quantified using the BioSpectrum 600 Imaging System (UVP, Upland, CA, USA).

### 2.5. Animals

The animal study was approved by the Institutional Animal Care and Use Committee (IACUC) of Industrial Technology Research Institute (ITRI; accredited by AAALAC) and was performed in accordance with the regulations of the Council of Agriculture, Taiwan (IACUC no. ITRI-IACUC-2017-056).

Eight-week-old male BALB/c mice were purchased from LASCO (Taipei, Taiwan). BALB/c mice have been widely used to establish IMQ-induced psoriasis-like mouse models. The animals were housed in the Animal Laboratory for Biomedical Research at ITRI in conventional cages under standardized conditions (23°C ± 2°C, 40%–70% relative humidity, positive pressure, ventilation rate of 15–20 changes/h, and a 12-hour light-dark cycle where the dark phase was from 7 : 30 pm to 7 : 30 am). The mice were allowed to acclimatize to their new environment for at least 1 week and were fed a standard chow diet and given water ad libitum.

### 2.6. IMQ-Induced Psoriasis-like Mouse Model

Before the experiment, the fur on the dorsal skin was shaved and treated with depilatory cream. IMQ (3.125 mg/mouse) was topically applied to an area of approximately 4 × 2 cm^2^ of the shaved skin once daily for 6 consecutive days. Pinocembrin was dissolved in 8% Olivem 1000 emulsifier. Concomitant with IMQ administration, the mice were topically administered pinocembrin (2.5 mg/mouse or 5 mg/mouse) once daily, whereas the mice treated with 8% Olivem 1000 emulsifier alone were used as the vehicle group. The mice were euthanized on day 6, and their dorsal skin was collected for histological and immunohistochemistry (IHC) examination.

### 2.7. Skin Scoring

The severity of the psoriatic dorsal skin was assessed using the Psoriasis Area and Severity Index. Clinical observation was performed daily. Erythema, the presence of scales (scaling), and thickening were evaluated on a scale from 0 to 4, with 0 representing none and 4 representing highly marked. The cumulative score (sum scores of erythema, scaling, and thickening) was the indicator of inflammation.

### 2.8. Histology and IHC

The mice were euthanized on day 6 after treatment with IMQ and pinocembrin. The lesional skin was fixed in 10% phosphate-buffered formaldehyde. Paraffin-embedded 3 *μ*m thick sections of the skin were stained with hematoxylin and eosin (H&E). Histopathological lesions of the skin were scored by a veterinary pathologist based on epidermal thickness, inflammation, hyperplasia, and hyperkeratosis. The sections were examined under an optical microscope (Leica DM2700 M, USA), and the thickness of the epidermal layer was evaluated in 3 random sections at 100× magnification by performing PolyChrome 1600 image analysis. The quantitative analysis of histopathological lesions on the skin was performed as per the reference method published in *Toxicologic Pathology* (Shackelfold et al., 2002). The severity of all microscopic lesions was graded as follows: 0 = not present; 1 = minimal (<1%); 2 = slight (1%–25%); 3 = moderate (26%–50%); 4 = moderately severe/high (51%–75%); and 5 = severe/high (76%–100%). For IHC, the paraffin-embedded sections were deparaffinized, gradually dehydrated, and treated with 0.5% H_2_O_2_ to block endogenous peroxidase activity. The sections were incubated with 10% normal goat serum and primary antibodies (anti-mouse CD4 antibody at 1/1000 dilution, anti-mouse CD8 antibody at 1/200 dilution, and anti-mouse Ki-67 antibody at 1/100 dilution), followed by incubation with the HRP-conjugated goat anti-rabbit secondary antibody (at 1/500 dilution). Immunopositive infiltrating cells were detected under an optical microscope (Leica DM2700 M, USA).

### 2.9. Real-Time Quantitative Polymerase Chain Reaction

Total RNA was isolated from HaCaT cells or IMQ-treated mouse skin by using the Qiagen RNeasy mini kit. Total RNA (2 *μ*g) was reverse-transcribed to cDNA by using the QuantiTect reverse transcription kit on the GeneAmp PCR System 9700. Real-time quantitative polymerase chain reaction (RT-qPCR) amplification of cDNA aliquots was performed using SYBR Green Supermix on the CFX connect real-time PCR system by using the following sense and antisense primers.

#### 2.9.1. Mouse Primers

Mouse primers are as follows: IL-1*β*, F-5′TCGTGCTGTCGGACCCAT3′ and R-5′TTGTTGGTTGATATTCTGTCCATTG3′; IL-6, F-5′TGCCATTGCACAACTCTTTTCT3′ and R-5′TCGGAGGCTTAATTACACATGTTC3′; IL-17, F-5′GCTCCAGAAGGCCCTCAGACT3′ and R-5′CCAGCTTTCCCTCCGCATTGA3′; IL-22, F-5′GAAGGCTGAAGGAGACAGTGAAA3′ and R-5′GTTCCCCAATCGCCTTGA3′; IL-23, F-5′GTATCCAGTGTGAAGATGGTTGTGA3′ and R-5′CGGATCCTTTGCAAGCAGAA3′; HO-1, F-5′CCTTCCCGAACATCGACAGCC3′ and R-5′GCAGCTCCTCAAACAGCTCAA3′; K16, F-5′AACAGCCTAGAAGAGACCAAAGGC3′ and R-5′GGTAGGGGAGACAGATGGGGAATGCGC3′; K17, F-5′GTGACCACCCGCCAGGTGCGC3′ and R-5′CAGTGTTCAGAACAAAGGCCACAG3′; and *β*-actin, F-5′TGGAATCCTGTGGCATCCATGAAAC3′ and R-5′TAAAACGCAGCTCAGTAACAGTCCG3′.

#### 2.9.2. Human Primers

Human primers are as follows: TNF-*α*, F-5′GGAGAAGGGTGACCGACTCA3′ and R-5′CTGCCCAGACTCGGCAA3′; IL-6, F-5′GTACCCCCAGGAGAAGATTC3′ and R-5′GCCATCTTTGGAAGGTTCAG3′; IL-17, F-5′ACTACAACCGATCCACCTCAC3′ and R-5′ACTTTGCCTCCCAGATCACAG3′; K16, F-5′GACCGGCGGAGATGTGAAC3′ and R-5′CTGCTCGTACTGGTCACGC3′; K17, F-5′GGTGGGTGGTGAGATCAATGT3′ and R-5′CGCGGTTCAGTTCCTCTGTC3′; Ki-67, F-5′CAAAGAGAGTGTCTATCAGCCG3′ and R-5′ACCAAGTTTTACTACATCTGCCC3′; and glyceraldehyde 3-phosphate dehydrogenase (GAPDH), F-5′AAGGTGAAGGTCGGAGTCAA3′ and R-5′AATGAAGGGGTCATTGATGG3′. The RNA expression level was normalized to that of the housekeeping gene *β*-actin for mice and GAPDH for humans, using the 2^−ΔΔ*C*t^ method.

### 2.10. Statistical Analysis

Data are presented as mean ± standard deviation (SD) or the mean ± standard error of the mean (SEM), as indicated in the figure legends. Intergroup differences were analyzed using Student's two-tailed *t*-test or the Mann–Whitney *U* test. All statistical analyses were performed using GraphPad Prism (version 8.4.3 for Windows, GraphPad Software, La Jolla, CA, USA). Statistical significance was defined as *p* < 0.05.

## 3. Results

### 3.1. Pinocembrin Reduces Inflammatory Cytokine Production and Cell Proliferation in Human Keratinocyte HaCaT Cells

**Figure 1:**
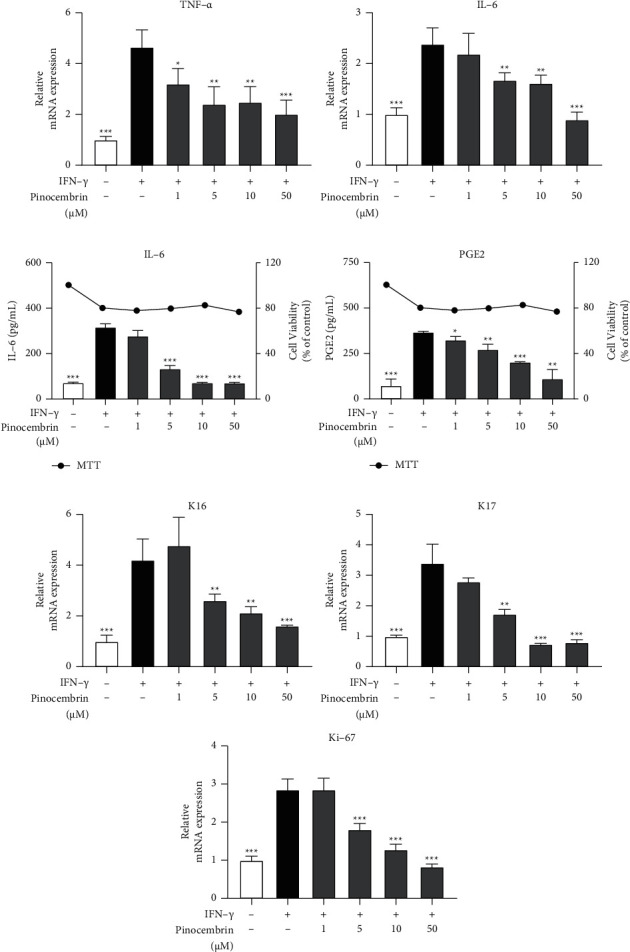
Anti-inflammatory and antiproliferative effects of pinocembrin on human keratinocytes. (a) HaCaT cells were pretreated with or without pinocembrin for 6 h and then cultured in the presence or absence of IFN-*γ* for 18 h; mRNA expression was quantified using qPCR. (b) Inflammatory cytokines and cell viability were determined using an ELISA and MTT assay, respectively. (c) HaCaT cells were pretreated with or without pinocembrin for 6 h and then cultured in the presence or absence of IFN-*γ* for 48 h; mRNA expression was quantified using qPCR. Data are expressed as mean ± SD. ^*∗*^*p* < 0.05, ^*∗∗*^*p* < 0.01, and ^*∗∗∗*^*p* < 0.001 compared with the IFN-*γ*-alone group, determined using Student's two-tailed *t*-test.

On the basis of the versatile biological activities of flavonoids, we investigated the pharmacological effects of pinocembrin on psoriasis. First, we examined the effects of pinocembrin on HaCaT cells. IFN-*γ* was chosen as an activator for HaCaT cells owing to the critical contribution of IFN-*γ* to the pathogenesis of psoriasis in humans. HaCaT cells were pretreated with pinocembrin and subsequently stimulated with IFN-*γ*. The results revealed that pinocembrin inhibited the mRNA expression of inflammatory cytokines induced by IFN-*γ*, including TNF-*α* (*F*-value = 1.502 and *p* = 0.0009 at 50 *μ*M) and IL-6 (*F*-value = 4.365 and *p* = 0.0001 at 50 *μ*M) (Figure 1(a)). Moreover, the protein levels of IL-6 (*F*-value = 66.92 and *p* < 0.0001 at 50 *μ*M) and PGE2 (*F*-value = 28.93 and *p* = 0.0015 at 50 *μ*M) were significantly decreased under the noncytotoxic condition (Figure 1(b)). After pinocembrin treatment, the keratinocyte hyperproliferation markers K16 (*F*-value = 271.6 and *p* = 0.0007 at 50 *μ*M), K17 (*F*-value = 47.9 and *p* = 0.0002 at 50 *μ*M), and Ki-67 (*F*-value = 13.83 and *p* < 0.0001 at 50 *μ*M) were all decreased considerably (Figure 1(c)). Collectively, these data demonstrated that pinocembrin could alleviate inflammatory responses and reduce keratinocyte hyperproliferation.

### 3.2. Pinocembrin Regulates the HO-1/STAT3 Pathway in HaCaT Cells

**Figure 2:**
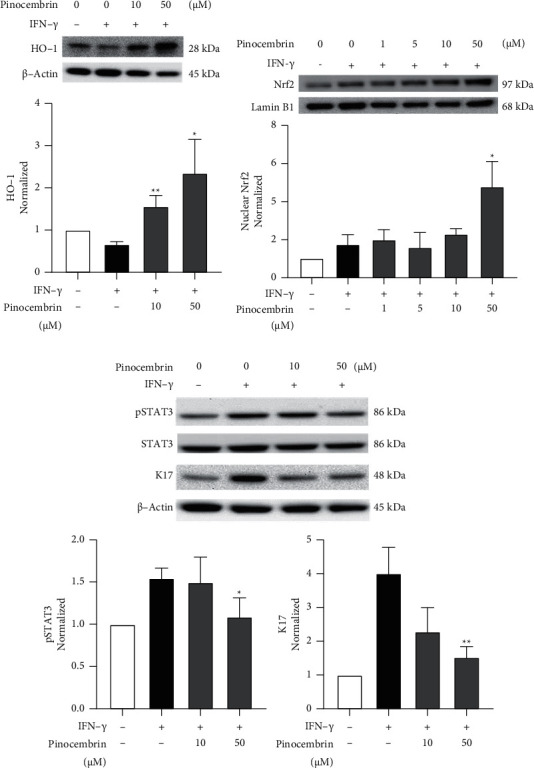
Effects of pinocembrin on Nrf2/HO-1, pSTAT3, and K17 expression in human keratinocytes. HaCaT cells were pretreated with or without pinocembrin for 6 h and then cultured in the presence or absence of IFN-*γ* for 48 h. The expression levels of (a) HO-1, (b) nuclear Nrf2, (c) pSTAT3, and K17 were examined through Western blotting. Data are expressed as mean ± SD. ^*∗*^*p* < 0.05 and ^*∗∗*^*p* < 0.01 compared with the IFN-*γ*-alone group, determined using Student's two-tailed *t*-test.

**Figure 3:**
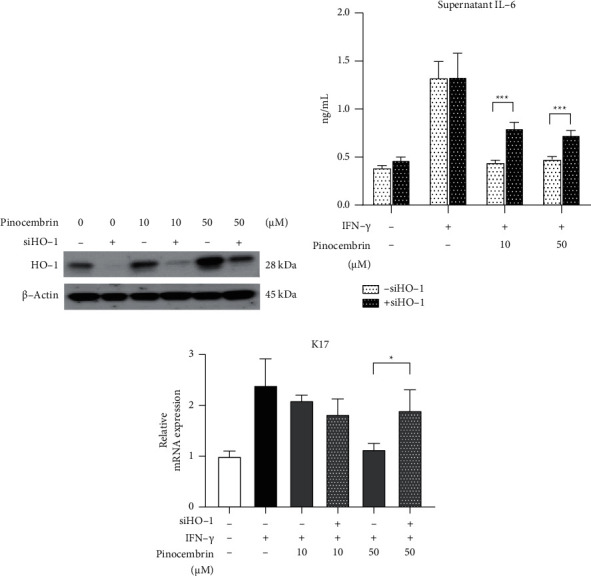
Knockdown of HO-1 reduced the efficacy of pinocembrin in HaCaT cells. (a) HaCaT cells were transfected with or without HO-1 siRNA for 6 h and then incubated with or without pinocembrin for 48 h; HO-1 expression was examined through Western blotting. (b) HaCaT cells were transfected with or without HO-1 siRNA for 6 h and then incubated with or without pinocembrin for 24 h in the presence or absence of IFN-*γ*. The cytokine IL-6 in the supernatant was analyzed through ELISA, and (c) the mRNA expression level of K17, a proliferation marker, was determined through qPCR. Data are expressed as mean ± SD. ^*∗*^*p* < 0.05 and ^*∗∗∗*^*p* < 0.001 compared with the non-siRNA-transfected group, determined using Student's two-tailed *t*-test.

HO-1 is a stress-induced antioxidative enzyme. A study reported that pinocembrin induced HO-l expression in neuroblastoma SH-SY5Y cells [27]. However, the effects of pinocembrin on HO-1 in the skin are not fully understood. Therefore, we examined the correlation between pinocembrin and HO-1 in HaCaT cells. As shown in Figure 2(a), pinocembrin significantly increased HO-1 expression at 10 (*F*-value = 19.99 and *p* = 0.0039) and 50 *μ*M (*F*-value = 195.3 and *p* = 0.0214). Furthermore, pinocembrin significantly increased the protein level of Nrf2 (*F*-value = 5.885 and *p* = 0.0226 at 50 *μ*M), which is the upstream master regulator of HO-1 (Figure 2(b)). Considering the substantial role of STAT3 in cytokine-activated keratinocytes, we investigated the effect of pinocembrin on STAT3. The results indicated that pSTAT3 expression was significantly attenuated by 50 *μ*M pinocembrin (*F*-value = 3.815 and *p* = 0.0367). In addition, the expression of K17, the hallmark of psoriatic keratinocytes, was also repressed when HaCaT cells were treated with 50 *μ*M pinocembrin (*F*-value = 5.884 and *p* = 0.0075) (Figure 2(c)). To further confirm the crucial role of HO-1 in the biological effects of pinocembrin, we knocked down HO-1 in HaCaT cells by using siRNA. The HO-1 expression induced by pinocembrin was remarkably decreased in HaCaT cells after the transfection of HO-1 siRNA (Figure 3(a)). When HO-1 expression was decreased, the inhibitory effects of pinocembrin on IL-6 secretion (*F*-value = 2.43 and *p* < 0.0001 at 50 *μ*M) and K17 mRNA expression were attenuated (*F*-value = 11.38 and *p* = 0.0113 at 50 *μ*M) (Figures 3(b) and 3(c)). These results demonstrated that pinocembrin reduced inflammation and keratinocyte proliferation through the upregulation of HO-1 and the downregulation of pSTAT3 in HaCaT cells.

### 3.3. Pinocembrin Ameliorates Skin Lesions in an IMQ-Induced Psoriasis-like Mouse Model

#### 3.3.1. IMQ-Induced Psoriasis-like Mouse Model

**Figure 4:**
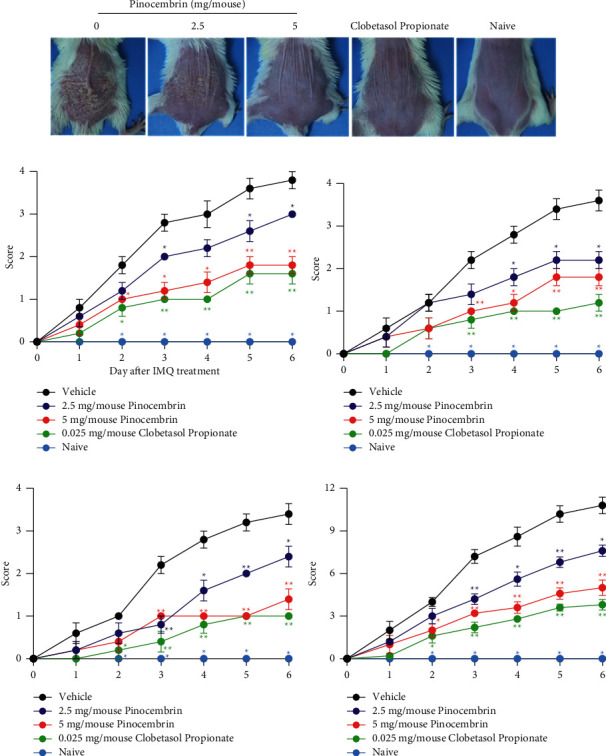
Pinocembrin ameliorated IMQ-induced skin lesions. (a) Representative images of skin tissue on day 6. (b) Erythema score, (c) thickness score, (d) scaling score, and (e) cumulative score (erythema + thickness + scaling) of skin tissue. BALB/c mice were topically administered Aldara cream (5% IMQ) on the dorsal skin for 6 consecutive days, and pinocembrin was concomitantly topically administered. Clobetasol propionate was used as a benchmark. Data are expressed as mean ± SEM (*n* = 5 for IMQ-treated animals and *n* = 3 for Naive). ^*∗*^*p* < 0.05 and ^*∗∗*^*p* < 0.01 compared with the vehicle group at the same day, determined using the Mann–Whitney *U* test.

IMQ is an agonist of TLR7 and TLR8 and is approved for the treatment of genital warts, superficial basal cell carcinoma, and actinic keratosis. Clinical observations have revealed that the topical application of Aldara cream (5% IMQ) caused psoriasis in the treated area and at distinct sites in patients with superficial basal cell carcinoma [28, 29]. Accordingly, the preclinical IMQ-induced dermatitis mouse model was established, and it has become the representative model for studying the pathogenesis of psoriasis. Therefore, we used an IMQ-induced mouse model to evaluate the biological effects of pinocembrin. IMQ was topically applied to the shaved dorsal skin of BALB/c mice for 6 consecutive days, and the severity of psoriasis-like skin conditions characterized by erythema, scaling, and thickness was scored daily. As expected, we observed that, compared with vehicle treatment, the topical administration of pinocembrin more significantly reduced cutaneous symptoms. The cumulative score on day 6 was 10.8 ± 0.6 for the group treated with the vehicle but 5.0 ± 0.6 for the group treated with 5 mg of pinocembrin, indicating a 54% alleviation of symptoms (*p* = 0.0079) (Figure 4).

#### 3.3.2. Histopathology and IHC Analysis

**Figure 5:**
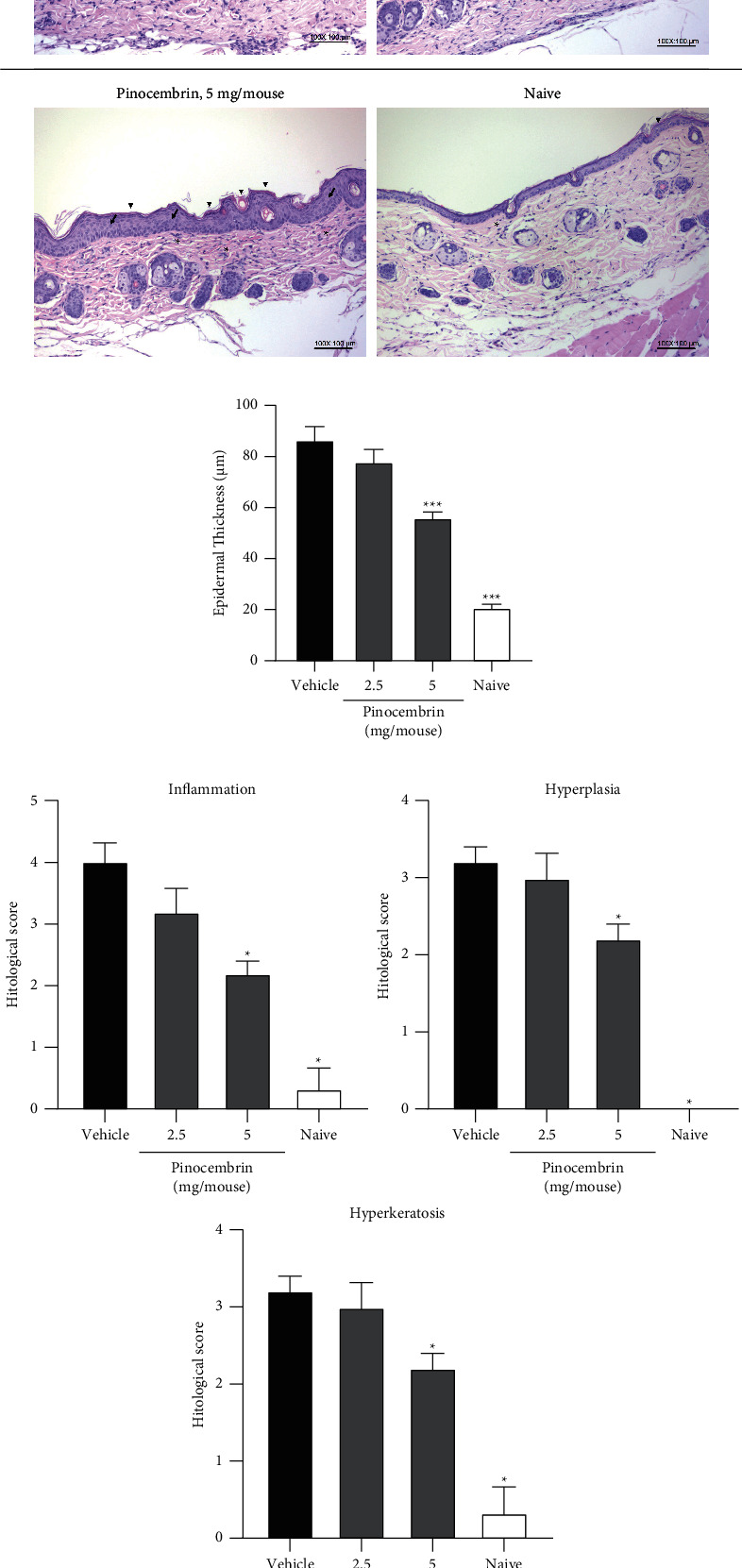
Histopathological analysis of mouse dorsal skin. (a) H&E stain (magnification 100×) ∗: inflammation. Arrow: hyperplasia. Arrowhead: hyperkeratosis. (b) Epidermal thickness. (c) Semiquantitative recording of histopathological lesions. On day 6 after IMQ challenge, mouse dorsal skin samples were harvested and then subjected to H&E staining. Inflammation, hyperplasia, and hyperkeratosis were semiquantified as described in Materials and Methods. Data are expressed as mean ± SEM (*n* = 5 for IMQ-treated animals and *n* = 3 for Naive). ^*∗*^*p* < 0.05 and ^*∗∗∗*^*p* < 0.001 compared with the vehicle group, determined using Student's two-tailed *t*-test (for epidermal thickness) or the Mann–Whitney *U* test (for the histopathological score).

**Figure 6:**
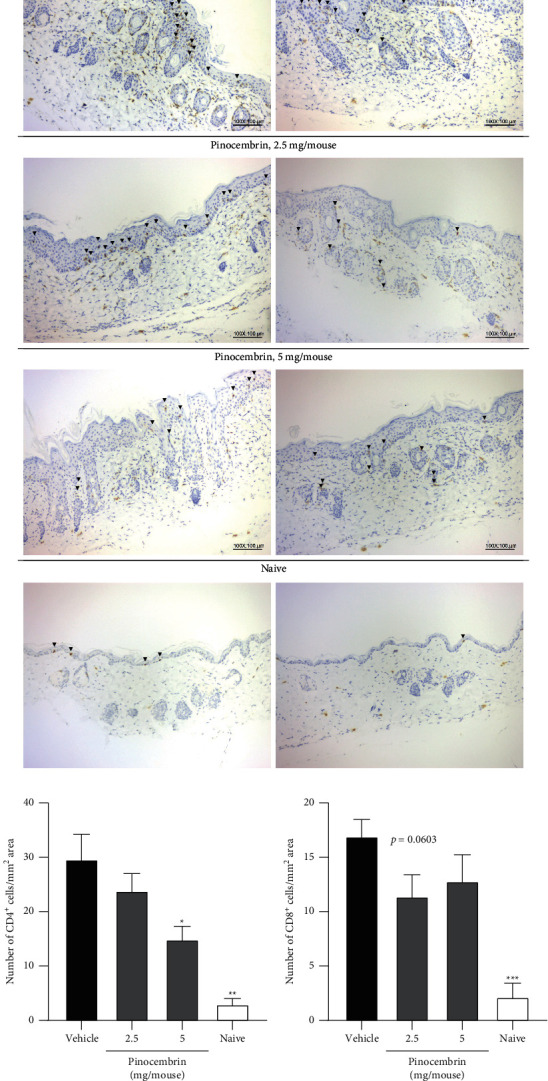
Pinocembrin reduced CD4^+^ and CD8^+^ T-cell infiltration in the IMQ-treated skin. (a) IHC analysis. Arrowhead: CD4^+^ or CD8^+^ T-cell infiltration. Quantitation of (b) CD4^+^ T-cells and (c) CD8^+^ T-cells. On day 6 after IMQ challenge, the infiltrated T-cells in the lesional skins were quantified through IHC analysis by using specific antibodies. The numbers of CD4^+^ and CD8^+^ cells were determined using an optical microscope. Data are expressed as mean ± SEM (*n* = 5 for IMQ-treated animals and *n* = 3 for Naive). ^*∗*^*p* < 0.05, ^*∗∗*^*p* < 0.01, and ^*∗∗∗*^*p* < 0.001 compared with the vehicle group, determined using Student's two-tailed *t*-test.

The H&E staining of the dorsal skin was performed to confirm the efficacy of pinocembrin, and the results were consistent with the observation of morphological features (Figure 5(a)). We found that the epidermal thickness significantly decreased in the group treated with 5 mg of pinocembrin compared with the vehicle group (55.91 ± 7.97 *μ*m versus 86.47 ± 13.14 *μ*m, *F*-value = 5.23 and *p* = 0.0007 at 50 *μ*M; Figure 5(b)). Furthermore, inflammation, hyperplasia, and hyperkeratosis were alleviated in the mice treated with 5 mg of pinocembrin (*p* = 0.0159 for inflammation; *p* = 0.0397 for hyperplasia; *p* = 0.0397 for hyperkeratosis; Figure 5(c)). We performed the IHC evaluation of the IMQ-treated skin to analyze the degree of T-cell infiltration. Our results revealed that pinocembrin inhibited CD4^+^ T-cell (*F*-value = 3.994 and *p* = 0.0261 at 5 mg) and CD8^+^ T-cell infiltration (*F*-value = 1.653 and *p* = 0.0603 at 2.5 mg) into the inflamed skin (Figure 6).

#### 3.3.3. Spleen Weight and Skin Cytokines

**Figure 7:**
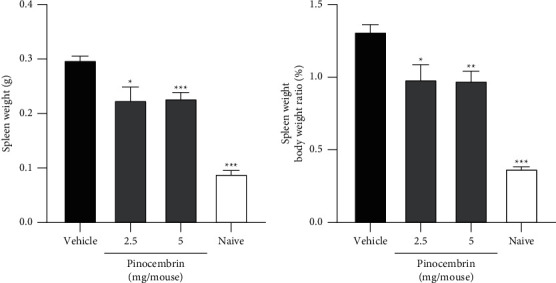
Effects of pinocembrin on spleen weight and spleen weight to body weight ratio. (a) Spleen weight. (b) Spleen weight to body weight ratio. On day 6 after IMQ challenge, the mice were euthanized, and the spleen weights and body weights were recorded. Data are expressed as mean ± SEM (*n* = 5 for IMQ-treated animals and *n* = 3 for Naive). ^*∗*^*p* < 0.05, ^*∗∗*^*p* < 0.01, and ^*∗∗∗*^*p* < 0.001 compared with the vehicle group, determined using Student's two-tailed *t*-test.

**Figure 8:**
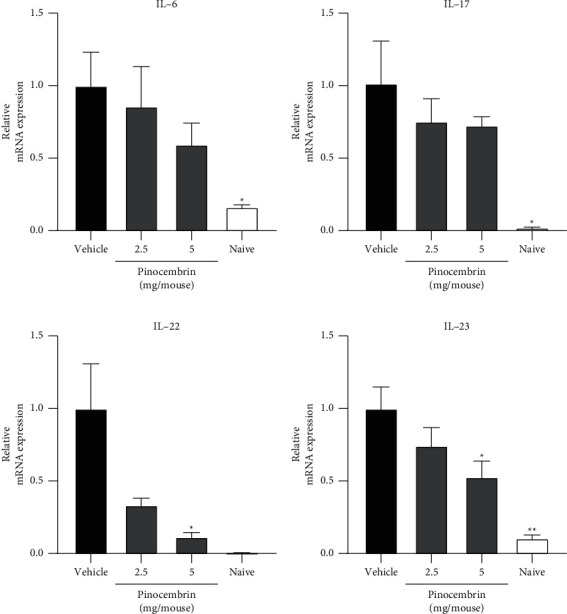
Pinocembrin ameliorated inflammatory cytokine production in IMQ-treated mice. (a) IL-6, (b) IL-17, (c) IL-22, and (d) IL-23 mRNA expression. Mice were challenged with IMQ for 6 consecutive days and euthanized on day 6. Skin tissues from IMQ-treated dorsal skins were harvested, and cytokine mRNA expression levels were quantified through qPCR. Data are expressed as mean ± SEM (*n* = 5 for IMQ-treated animals and *n* = 3 for Naive). ^*∗*^*p* < 0.05 and ^*∗∗*^*p* < 0.01 compared with the vehicle group, determined using Student's two-tailed *t*-test.

The enlargement of the spleen is a characteristic of IMQ-induced skin inflammation in mice. The spleen weight was over 3-fold greater in the vehicle group than in the treatment-naïve group. Both spleen weight and spleen weight to body weight ratio were significantly decreased in the pinocembrin-treated groups compared with the vehicle group (*F*-value = 2.412 and *p* = 0.0005 for spleen weight at 5 mg; *F*-value = 1.826 and *p* = 0.0031 for spleen weight to body weight ratio at 5 mg; Figure 7). We subsequently examined cytokine expression in the skin samples. The results demonstrated that the mRNA levels of psoriasis-related cytokines, namely, IL-22 and IL-23, were significantly decreased in the group treated with 5 mg of pinocembrin compared with the vehicle-treated group (*F*-value = 101 and *p* = 0.0209 for IL-22; *F*-value = 1.83 and *p* = 0.0338 for IL-23; Figure 8). The mRNA levels of IL-6 and IL-17 were lower in the pinocembrin-treated group, but the differences were not statistically significant (Figure 8).

#### 3.3.4. Pinocembrin Alters the Expressions of HO-1, pSTAT3, and Keratinocyte Activation Markers in IMQ-Treated Mice

**Figure 9:**
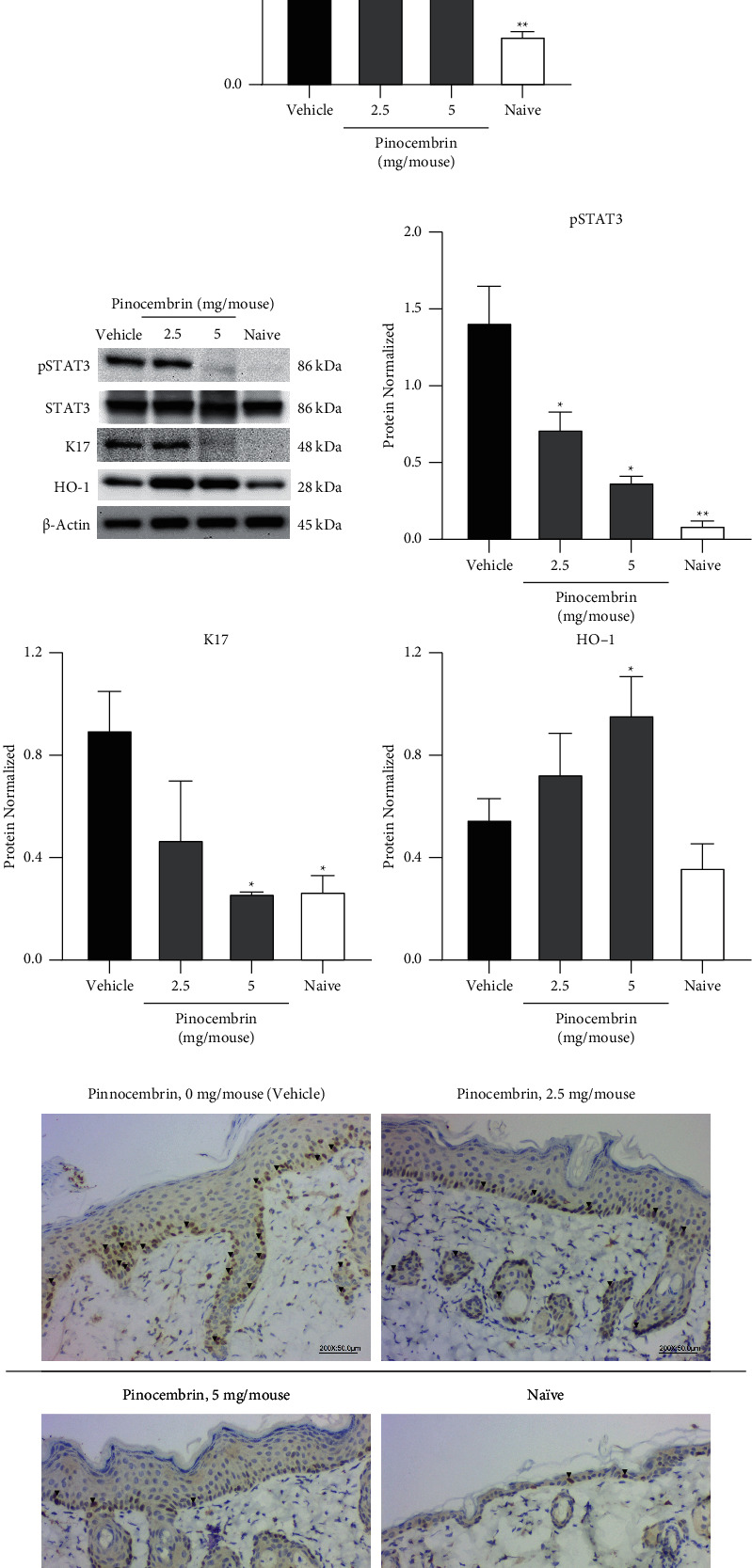
Pinocembrin affected HO-1, K16, K17, pSTAT3, and Ki-67 expression in IMQ-treated mouse skin. Mice were challenged with IMQ for 6 consecutive days and euthanized on day 6. Skin tissues from IMQ-treated dorsal skins were harvested and then subjected to (a) qPCR, (b) Western blotting, and (c) IHC analysis. Arrowhead: Ki-67^+^ cells. Data are expressed as mean ± SEM (*n* = 3–5 for IMQ-treated animals and *n* = 3 for Naive). ^*∗*^*p* < 0.05, ^*∗∗*^*p* < 0.01, and ^*∗∗∗*^*p* < 0.001 compared with the vehicle group, determined using Student's two-tailed *t*-test.

On the basis of the finding that pinocembrin affected HO-1, pSTAT3, and K17 expression in HaCaT cells in vitro, we evaluated the effects of pinocembrin on these markers in the IMQ-treated mouse skin. The results indicated that, compared with the vehicle-treated group, the pinocembrin-treated group exhibited upregulated mRNA expression of HO-1 (*F*-value = 15.03 and *p* < 0.0001 at 5 mg) but downregulated expression of the keratinocyte proliferation markers K16 (*F*-value = 5.156 and *p* = 0.0006 at 5 mg) and K17 (*F*-value = 2.408 and *p* = 0.0112 at 5 mg) (Figure 9(a)). Moreover, the results of Western blot analysis revealed that pinocembrin induced HO-1 expression (*F*-value = 2.673 and *p* = 0.049 at 5 mg) but inhibited pSTAT3 (*F*-value = 58.33 and *p* = 0.0136 at 5 mg) and K17 expression (*F*-value = 440.2 and *p* = 0.0151 at 5 mg) (Figure 9(b)). Furthermore, the IHC evaluation demonstrated that the keratinocyte proliferation marker Ki-67 was significantly downregulated in the pinocembrin-treated skin compared with the vehicle-treated group (*F*-value = 3.015 and *p* = 0.0021 at 5 mg; Figure 9(c)). Cumulatively, the in vivo results are consistent with the in vitro findings.

## 4. Discussion

Psoriasis is a chronic, prevalent, noncommunicable, autoimmune disease. This disease can occur in a person of any age and ethnicity and affects more than 125 million people worldwide [1]. The presence of skin lesions on highly visible areas such as the face, hands, and legs can impede the daily social activities of patients with psoriasis. In addition, systemic inflammation caused by psoriasis can result in several comorbidities such as cardiovascular disease, metabolic syndrome, and nonalcoholic steatohepatitis [30]. Therefore, affected patients frequently experience anxiety, depression, and suicidal ideation and may have poor quality of life [31]. No absolute cure is available for psoriasis, and demand for a new therapeutic drug for psoriasis persists.

The crosstalk between keratinocytes and immune cells can contribute to chronic inflammation in psoriasis [32]. Keratinocytes play substantial roles in initiating and orchestrating innate and adaptive immune responses. The antimicrobial peptide LL37, which is derived from keratinocytes, forms a complex with extracellular self-nucleic acids and initiates an innate immune response. Hence, LL37-nucleic acid complexes enable the activation of plasmacytoid dendritic cells; maturation of myeloid dendritic cells; and further production of type I IFN, IL-6, and TNF-*α*, thus driving T-cell activation. Furthermore, the findings of histopathology analysis demonstrated that LL37+ keratinocytes were coexpressed with macrophages, dendritic cells, and T-cells in the dermis of lesional psoriatic skin [33]. In addition, a genetic study reported that the mutation of CARD14, which is mainly expressed in keratinocytes, may trigger psoriasis by NF-*κ*B activation [34]. On the basis of these findings, keratinocytes are considered the essential driver of psoriasis.

Keratin is a cytoskeletal filament protein that provides mechanical support and enables keratinocytes to maintain epidermal integrity. Studies have reported that the aberrant expression of K6, K16, and K17 in the psoriatic skin is regulated by the transcription factor Nrf2, and K6, K16, and K17 are considered the hallmarks of keratinocyte activation [13, 35]. Notably, K17 is not typically observed in healthy skin and is only expressed in psoriatic lesions. Moreover, K17 is typically an autoantigen for psoriatic T cells, and it has hyperproliferation-associated function. The activation of keratinocytes by Th1- and Th17-derived cytokines might lead to the overexpression of K17. Subsequently, psoriatic T-cells are activated by epitopes contained within K17 and then release abundant inflammatory cytokines to activate more keratinocytes. This vicious cycle leads to chronic inflammation in psoriasis [36].

Although considerable focus has been placed on the effect of IL-17 in the psoriatic environment, IFN-*γ* remains the central tenet in the pathogenesis of psoriasis. Studies have reported that the IFN-*γ* level was increased in the serum of patients with psoriasis and that IFN-*γ* was important for the activation of IL-17(+) T-cells. Moreover, K17 was overexpressed in HaCaT cells under IFN-*γ* stimulation [37–39]. Concordant with these findings, our results revealed that IFN-*γ* induced HaCaT cell activation and pinocembrin reduced keratin expression and psoriatic cytokine secretion in IFN-*γ*-stimulated HaCaT cells (Figure 1).

HO-1 is an inducible metabolic enzyme responsible for the degradation of heme. In addition to this metabolic function, several studies have revealed that the cytoprotective functions of HO-1 include antioxidative and anti-inflammatory activities. Furthermore, the upregulation of HO-1 is correlated with tumor growth, metastasis, angiogenesis, and drug resistance, thereby making HO-1 a novel drug target for cancer therapy [40]. Previous research regarding psoriasis has indicated that HO-1 was strongly overexpressed in human psoriatic skin and lesional skin in an IMQ-induced psoriatic mouse model [18, 41]. Moreover, under oxidative stress, 2 sesquiterpene aminoquinones, namely, dysidaminone H and 3′-methylamino-avarone, exerted a cytoprotective effect on HaCaT cells through the upregulation of the Nrf2/HO-1 pathway [42]. Furthermore, natural HO-1 inducers, namely, curcumin and carnosol, limited dendritic cell maturation, and curcumin further inhibited T-cell activation in psoriatic peripheral blood mononuclear cells ex vivo [43]. In a di-n-propyl disulfide-induced psoriatic-like mouse model, the topical administration of propolis, epigallocatechin 3-gallate, curcumin, quercetin, and chrysin alleviated lesions and macrophage infiltration into the peritoneal cavity [44]. These findings suggest that different flavonoids possess HO-1 regulatory activity and can be beneficial for psoriasis. Therefore, we examined the pharmacological activity of pinocembrin in an IMQ-induced psoriasis-like mouse model. The results revealed that pinocembrin ameliorated psoriatic dermatitis, and its underlying mechanism of action involved the regulation of HO-1 (Figures 4 and 9).

The transcription factor STAT3 is the central regulator of various immune responses. STAT3 conveys signals from psoriatic cytokines, such as IL-6, IL-17, IL-21, IL-22, and IL-23, to the nucleus and triggers an inflammatory response, which is involved in the pathogenesis of psoriasis [20, 45]. Previous research has demonstrated that the anticancer drug sunitinib, the phytochemicals shikonin and cryptotanshinone, and the herbal isoflavone extract reduced epidermal keratinocyte activation through the regulation of STAT3 [46–49]. Notably, a clinical study reported that a STAT3 inhibitor improved the skin lesions of patients with psoriasis and indicated that STAT3 can serve as a drug target for psoriasis [50]. In this study, we observed that pinocembrin upregulated HO-1 expression and simultaneously downregulated STAT3 activation (Figure 9). These data indicate the therapeutic potential of pinocembrin for psoriasis.

More and more studies have shown that the Th17 cells are associated with autoimmune diseases, such as psoriasis, multiple sclerosis, and rheumatoid arthritis [51–54]. Inflammatory cytokine IL-6, TGF-*β*, and transcriptional factor STAT3 are required for Th17 cells differentiation, and IL-23 is essential for Th17 cells expansion. Following the activation, Th17 cells produce IL-17 and IL-22 which contribute to the inflammatory response in autoimmune diseases [55–57]. In this study, our data showed that pinocembrin ameliorated cytokines IL-6, IL-17, IL-22, and IL-23 expression and reduced STAT3 activation. These results implicated that pinocembrin may have an inhibitory effect of Th17 cells response. Further study is required to reveal the mechanism in detail.

Several studies have reported that psoriasis is one of the comorbidities of autism spectrum disorder (ASD) characterized by defeats of social function. Current reports have shown the upregulated Ki-67 in T-cells and overreactive innate and adaptive immune responses may contribute to ASD and psoriatic comorbidity of ASD [58, 59]. Since our studies showed that pinocembrin possessed Ki-67 inhibitory and anti-inflammatory activities, it is interesting to assess the therapeutic potential of pinocembrin for ASD and ASD-associated psoriasis in future study.

Flavonoids are a class of polyphenolic compounds widely found in plants and the human diet. More than 5000 flavonoids have been identified and grouped into 10 categories [60, 61]. Flavonoids exert anti-inflammatory, antioxidative, and anticarcinogenic effects and are considered beneficial for human health. Pinocembrin is a flavonoid isolated from many plants families including Piperaceae, Lauraceae, Zingiberaceae, and Asteraceae [22]. Several biological activities of pinocembrin have been reported in cellular and animal models including antiviral, neuroprotective, antiallergic, antidiabetic, nephropathic, and antibacterial activities [62–66]. Notably, the neuroprotective effects of pinocembrin have been reported in human trials; thus, pinocembrin is considered a potential drug for ischemic stroke. Relevant trial results for pinocembrin treatment have increased our interest in exploring the therapeutic application of pinocembrin in other human diseases.

To our knowledge, no study had evaluated the pharmacological activities of pinocembrin for psoriasis. Our results demonstrated the therapeutic potential of pinocembrin for psoriasis in a preclinical animal disease model. Furthermore, the pharmacokinetics, pharmacodynamics, and toxicology data of pinocembrin obtained from clinical trials for ischemic stroke indicated that pinocembrin is safer than other flavonoids.

Additional studies should be conducted to identify other cellular targets of pinocembrin and explore the biological activities of pinocembrin in other transgenic mouse models for psoriasis. Notably, pinocembrin possesses HO-1 regulatory activity, and HO-1 is associated with pulmonary and cardiovascular diseases [67, 68]. Therefore, studies investigating the potential of pinocembrin in these diseases may prove worthwhile.

## 5. Conclusions

Psoriasis is a noncontagious systemic autoimmune disease, and patients with psoriasis often have poor quality of life. However, no absolute cure for psoriasis exists, and novel treatment options for psoriasis are urgently required. In this study, we observed that pinocembrin alleviated psoriatic symptoms in an IMQ-induced dermatitis mouse model. The mechanism of action involved the regulation of the HO-1/STAT3 pathway. Thus, pinocembrin warrants further investigation to establish its clinical benefits for patients with psoriasis.

## Data Availability

The data used to support the findings of this study are included within the article.
